# A Genome-Wide Homozygosity Association Study Identifies Runs of Homozygosity Associated with Rheumatoid Arthritis in the Human Major Histocompatibility Complex

**DOI:** 10.1371/journal.pone.0034840

**Published:** 2012-04-20

**Authors:** Hsin-Chou Yang, Lun-Ching Chang, Yu-Jen Liang, Chien-Hsing Lin, Pei-Li Wang

**Affiliations:** 1 Institute of Statistical Science, Academia Sinica, Taipei, Taiwan; 2 Graduate Institute of Biomedical Electronics and Bioinformatics, National Taiwan University, Taipei, Taiwan; 3 Division of Molecular and Genomic Medicine, National Health Research Institutes, Miaoli County, Taiwan; University of Hong Kong, Hong Kong

## Abstract

Rheumatoid arthritis (RA) is a chronic inflammatory disorder with a polygenic mode of inheritance. This study examined the hypothesis that runs of homozygosity (ROHs) play a recessive-acting role in the underlying RA genetic mechanism and identified RA-associated ROHs. Ours is the first genome-wide homozygosity association study for RA and characterized the ROH patterns associated with RA in the genomes of 2,000 RA patients and 3,000 normal controls of the Wellcome Trust Case Control Consortium. Genome scans consistently pinpointed two regions within the human major histocompatibility complex region containing RA-associated ROHs. The first region is from 32,451,664 bp to 32,846,093 bp (−log10(p)>22.6591). RA-susceptibility genes, such as *HLA-DRB1*, are contained in this region. The second region ranges from 32,933,485 bp to 33,585,118 bp (−log10(p)>8.3644) and contains other *HLA-DPA1* and *HLA-DPB1* genes. These two regions are physically close but are located in different blocks of linkage disequilibrium, and ∼40% of the RA patients' genomes carry these ROHs in the two regions. By analyzing homozygote intensities, an ROH that is anchored by the single nucleotide polymorphism rs2027852 and flanked by *HLA-DRB6* and *HLA-DRB1* was found associated with increased risk for RA. The presence of this risky ROH provides a 62% accuracy to predict RA disease status. An independent genomic dataset from 868 RA patients and 1,194 control subjects of the North American Rheumatoid Arthritis Consortium successfully validated the results obtained using the Wellcome Trust Case Control Consortium data. In conclusion, this genome-wide homozygosity association study provides an alternative to allelic association mapping for the identification of recessive variants responsible for RA. The identified RA-associated ROHs uncover recessive components and missing heritability associated with RA and other autoimmune diseases.

## Introduction

Rheumatoid arthritis (RA, OMIM #180300), characterized by damage to the synovial joints, is a chronic inflammatory disorder with a multifactorial etiology and a polygenic mode of inheritance [1], [2], [3], [4], [5]. RA patients have a shorter life expectancy (by ∼3–10 years) and a higher mortality rate (∼1.5–1.6 fold) compared with the general population [6], [7]. The worldwide prevalence rates and sibling-relative risks of RA are ∼0.3%–1.2% and 5–10-fold greater, respectively [8], [9]. Despite differences in the genetic backgrounds of RA patients, the significance of the *HLA-DRB1*-shared epitope (6p21.3) [10], [11], [12], [13] and *PTPN22* (1p13.3-p13.1) [14], [15], [16], [17], [18], [19], [20] in the genomes of RA patients has been well replicated in different genetic studies.

With the availability of high-throughput genotyping techniques, genome-wide single nucleotide polymorphism (SNP) arrays (e.g., Affymetrix GeneChip and Illumina BeadChip technologies) have been well developed and broadly applied to identify SNPs/genes associated with complex diseases [21]. Large-scale genome-wide association studies of RA have been carried out [17], [18], [20]. These studies, which have been replicated, identified several new RA-associated genes, including *TRAF1*/*C5* (9q33-q34) [17], [19], [22], *TNFAIP3* (6q23) [18], [19], [23], and *CTLA4* (2q33) [18], [19], [20]. Additional RA-associated genes have been found, which are pending confirmation, including *STAT4* (2q32.2–32.3) [24], *CD40* (20q13) [19], *REL* (2p13-p12) [25], *PRKCQ* (10p15) [26], and *PADI4* (1p36.13) [16]. Although previous studies have established allelic associations between RA and certain genomic regions, all of the genes that contribute to RA have not been found [27], i.e., >68% of the genetic variation responsible for RA remains to be identified [20]. Genome-wide homozygosity association mapping provides an alternative to allelic association mapping for identification of recessive-acting susceptibility genes, uncovering missing heritability, and understanding the complex etiological mechanism(s) of RA.

A run of homozygosity (ROH) denotes a contiguous set of homozygous genotypes in an intact genomic region. A practically used definition of ROH allows a rich homozygote region interrupted by a small number heterozygous genotypes arising from genotyping errors, missing genotypes, or mutations. An ROH that includes a sizable tract of homozygosity and deviates from a random distribution in the genome is denoted as “homozygosity disequilibrium” in this study. This type of ROH may result from various mechanisms including: 1) chromosomal aberrations, (e.g., uniparental disomy, hemizygous deletion, and/or loss of heterozygosity [28], [29], [30], [31], [32]); 2) autozygosity as a consequence of inbreeding, consanguineous marriage, or a recent common ancestor [33], [34], [35], [36], [37]; and 3) natural selection, e.g., positive selection or selective sweep [38], [39], [40]. Homozygosity disequilibrium has frequently been observed in the general outbred population [34], [41], [42], but it is also not entirely benign as it increases the susceptibility to diseases such as neurodevelopment-related disorders [40], [43] and other autoimmune diseases [44].

Homozygosity mapping aims to identify ROH(s) associated with disease states and was originally developed to map genes responsible for recessive diseases by using genetic marker data from inbred pedigrees [45], [46], [47], [48], [49]. Recent studies have also showed that homozygosity association mapping is a statistically powerful method when identifying susceptibility genes associated with complex diseases [40], [43], cancers [50], [51], [52], [53], and phenotypic traits [54], [55], [56]. Various statistical methods of homozygosity association mapping have been developed in order to analyze genotype data [35], [53], [57], [58], [59] or fluorescence intensity data [60], [61], [62], [63] from SNP microarrays. To the best of our knowledge, however, studies have not been performed for genome-wide homozygosity association mapping for RA. Additionally, ROHs have not been used as genetic markers for the prediction of RA status. Instead of focusing on allelic association as have previous genome-wide association studies for RA [17], [18], [20], this study examined the hypothesis that ROHs act as recessive-acting determinants in the underlying genetic mechanisms of RA and identified RA-associated ROHs using genome-wide homozygosity association mapping.

## Results

### Power calculations

**Figure 1:**
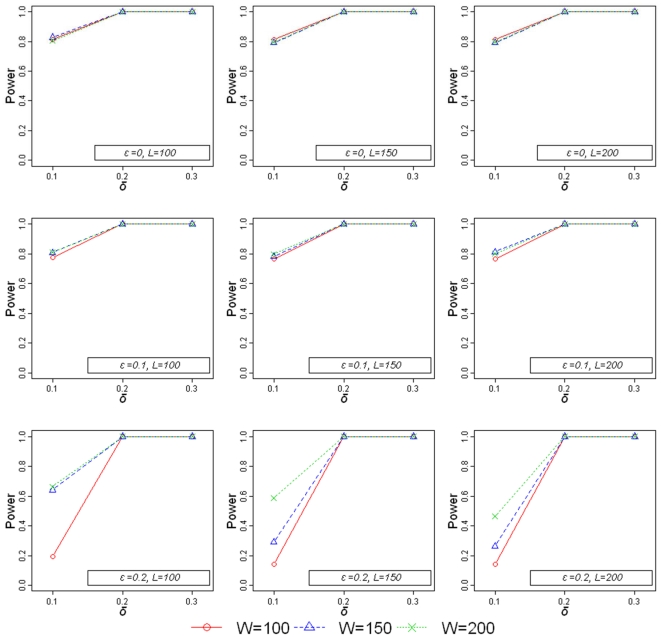
Calculated powers for simulated genome-wide homozygosity association scans. The simulated genome-wide homozygosity association scans included 2,000 patients and 3,000 controls and used −log_10_(p)>8 for the threshold of statistical significance. Parameters for the calculations included: The proportion of patients carrying the ROH (*δ* = 0.1, 0.2, or 0.3) controlled the effective size of a scan. The number of evenly spaced SNPs (*L* = 100, 150, or 200 SNPs) determined the length of the true disease-associated ROH. The heterozygous interference was defined as the fraction of heterozygous-call SNPs in the true disease-associated ROH (*ε* = 0, 0.1, or 0.2). *W* = 100, red solid line, circles; *W* = 150, blue dashed line, triangles; *W* = 200, green dotted line, crosses.

Based on the simulation procedures described in Appendix S1, values for the powers of simulated genome-wide homozygosity association mappings were calculated using 2,000 patients and 3,000 controls in a simulation study of 1,000 replications (Figure 1). We always used a genome-wide significance level of −log_10_(p)>8. First, we considered the scenario for which a disease-associated ROH consisted of *L* consecutive SNPs (*L* = 200). When 30%, 20%, and 10% of the RA patients carried this ROH (effect size, *δ*,  = 0.3, 0.2, 0.1), the power needed to detect the ROH was calculated as 1.000, 1.000, and 0.814, respectively, for a genome scan using a window size (*W*) of 100 SNPs (*W* = 100), or calculated as 1.000, 1.000, and 0.790, respectively, for *W* = 150, or as 1.000, 1.000, and 0.795, respectively, for *W* = 200. We also incorporated a heterozygous interference value (*ε*), as a fraction that denoted incomplete homozygosity in the disease-associated ROH that may be caused by genotyping errors or unknown mutation mechanisms. The power required for no heterozygous interference was very similar to the power required for 10% heterozygous interference. When *ε* = 0.2 and *δ* = 0.3 or 0.2, the power was 1.000. However, the power was reduced to 0.141 for a genome scan with *W* = 100, reduced to 0.263 for *W* = 150, and to 0.463 for *W* = 200 (when *ε* = 0.2 and *δ* = 0.1). We also considered a disease-associated ROH for *L* = 150 or 100 and found the powers to be very similar to that found for *L* = 200.

### Genome-wide homozygosity association scans

**Figure 2:**
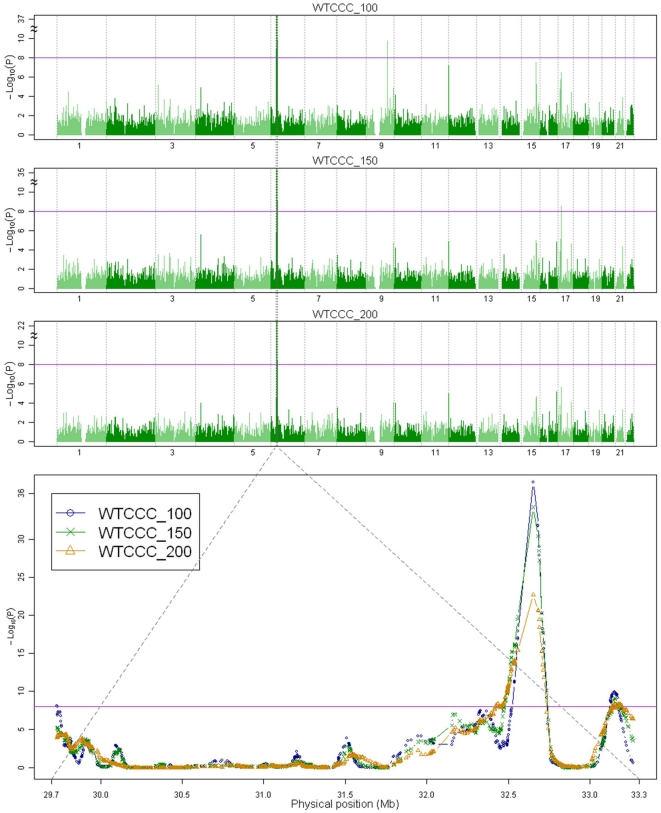
Genome-wide homozygosity association scans. The values of −log_10_(p) at the anchor SNPs for the three genome-wide homozygosity association scans, WTCCC_100, WTCCC_150, and WTCCC_200, are displayed. The purple, horizontal reference lines indicate −log_10_(p) = 8, the cut-off used to test for significance. Two peaks with −log_10_(p)>8 in the MHC region on chromosome 6p21.3 were found for all three scans. The bottom panel shows an expanded plot containing the region of the two peaks. WTCCC_100, blue line, circles; WTCCC_150, green line, crosses; WTCCC_200, orange line, triangles.

We conducted genome-wide homozygosity association scans with *W* = 100, 150, and 200 for the Wellcome Trust Case Control Consortium (WTCCC) SNP data (WTCCC_100, WTCCC_150, and WTCCC_200, respectively). Each genomic scan identified ROHs that satisfied the genome-wide significance criterion of −log_10_(p)>8 (Figure 2). The identified regions and the respective maximum values of −log_10_(p) within the identified regions are as follows. The WTCCC_100 scan identified three regions on chromosome 6p [−log_10_(p) = 8.0769, −log_10_(p) = 37.5332, and −log_10_(p) = 9.8852] and one region on 9q [−log_10_(p) = 9.7484]. The WTCCC_150 scan identified two regions on chromosome 6p [−log_10_(p) = 34.2091 and −log_10_(p) = 9.0952] and one region on 17p [−log_10_(p) = 8.5038]. The WTCCC_200 scan identified two neighboring regions on 6p [−log_10_(p) = 22.6591 and −log_10_(p) = 8.3644]. All three scans identified two overlapping ROHs located on chromosome 6p.

**Figure 3:**
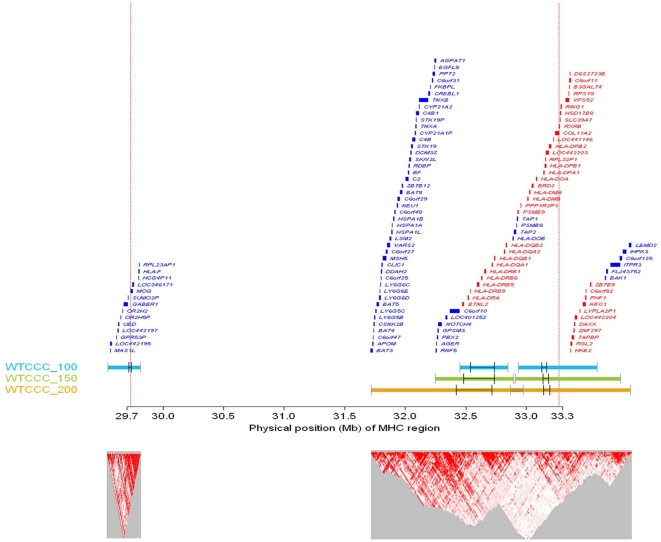
Genes and LD structures in the MHC region identified by the homozygosity association scans. The genes and intermarker LDs in the regions containing ROHs are displayed. The two dotted, red vertical lines demarcate the MHC region from 29,732,804 bp to 33,268,223 bp on chromosome 6p. The regions identified by the WTCCC_100, WTCCC_150, and WTCCC_200 scans are identified by the sky blue, light green, and orange horizontal bars, respectively. The outermost vertical bars denote the first SNP (gray tick) in the first window and the last SNP (gray tick) in the last window. Additionally, the first anchor SNP and the last anchor SNP for regions with −log_10_(p)>8 are marked using bold ticks. If two regions identified by the same genome scan overlap, the segment containing the overlapping regions is shown in dark blue, green, and orange for WTCCC_100, WTCCC_150, and WTCCC_200, respectively. The names of genes within the annotated regions are given above the bars. The names of genes in the regions identified by the three scans are shown in red, and the names of the genes identified by one or two scans are shown in blue. The location and width of the bars that prefix the gene names reflect the physical position and the size of the genes. LD structures are provided in the lower panels in which the higher intermarker LDs are in red.

The first of these overlapping ROHs ranged from 32,451,664 bp to 32,846,093 bp and is located within the human major histocompatibility complex (MHC) region at 6p21.3, and the second ranged from 32,933,485 bp to 33,585,118 bp and overlaps the MHC region (Figure 3). The two regions are located in different blocks of linkage disequilibrium (LD). The names of the genes within these two regions are shown in red in Figure 3. The first region contains 10 genes (from *BTNL2* to *HLA-DQB2*), and the number of SNPs and the average intermarker distance are 125 and 3.1554 kb, respectively. The maximum −log_10_(p) values for the scans are 37.5332 for WTCCC_100, 34.2091 for WTCCC_150, and 22.6591 for WTCCC_200. The second region contains 33 genes (from *PSMB9* to *ZBTB9*), and the number of SNPs and the average intermarker distance are 134 and 4.8629 kb, respectively.

The proportion that RA patients carried a specific ROH (pROH) is higher than in normal controls in the two regions of homozygosity disequilibrium. For sliding windows anchored by SNPs within the first region, the maximum number of pROHs, as a fraction, for the patient data is 0.2206 for WTCCC_100, 0.2331 for WTCCC_150, and 0.2071 for WTCCC_200. These values are greater than those of the controls: 0.0996 for WTCCC_100, 0.1003 for WTCCC_150, and 0.1003 for WTCCC_200. In the second region, the maximum −log_10_(p) values are 9.8852 for WTCCC_100, 9.0952 for WTCCC_150, and 8.3644 for WTCCC_200. The maximum number of pROHs is 0.1381 for WTCCC_100, 0.1331 for WTCCC_150, 0.1341 for WTCCC_200, and these figures are greater than the maximum number of pROHs for the normal control data (0.1003 for WTCCC_100, 0.1003 for WTCCC_150, and 0.1003 for WTCCC_200).

We investigated the correlation between the presence of these ROHs and RA disease status for anchoring SNPs within the two regions that satisfy −log_10_(p)>8 (Figure 3). The first region (ROH1) contains 26 anchor SNPs (rs9268831 to rs9273363) and five genes (*HLA-DRB9*, *HLA-DRB5*, *HLA-DRB6*, *HLA-DRB1*, and *HLA-DQA1*) (Figure S1). The second region (ROH2) contains 34 anchor SNPs (rs10807118 to rs7764491) and four genes (*HLA-DPA1*, *HLA-DPB1*, *RPL32P1*, and *LOC442203*) (Figure S1). A statistical discriminant analysis of 60 anchor SNPs (rs9268831 to rs7764491) showed that the highest average accuracy for predicting RA status is 0.6201 and is associated with SNP16 (rs2027852), which is flanked by *HLA-DRB6* and *HLA-DRB1*. The ROH anchored at rs2027852 was then used to predict RA status for the data from 868 RA patients and 1,194 controls of the North American Rheumatoid Arthritis Consortium (NARAC). The prediction accuracy is 0.5790.

The genetic heterogeneity of RA patients was investigated next. The distribution of pROH in RA patients at windows anchored by the 60 SNPs is shown in Figure S1. The pROH pattern suggests that there are three SNP groups (rs9268831 to rs7749092, rs2027852, and rs9270986 to rs9273363) in ROH1, and that there are two SNP groups (rs10807118 to rs3077 and rs9348904 to rs7764491) in ROH2. Within each SNP group, the pROH pattern is very similar. Therefore, only one “tag” anchor SNP was investigated further. These anchor SNPs are rs9268831 in *HLA-DRB9*, rs2027852 flanked by *HLA-DRB6* and *HLA-DRB1*, rs9272723 in *HLA-DQA1*, rs3077 in *HLA-DPA1*, and rs9277542 in *HLA-DPB1*. Thirty-two ROH-carrying categories for the patients were identified using the presence or absence of ROHs anchored by the five SNPs. Vectors made of five indicator variables describe the categories. When the value of the *i*th indicator was 1, the genomic segment anchored by the *i*th anchor SNP carried an ROH; otherwise, the value of the indicator was 0. Seven of the categories have a pROH >2% in RA patients; the pROHs are P(0,0,0,0,0) = 60.13%, P(1,0,0,0,0) = 7.5%, P(1,1,1,0,0) = 6.85%, P(0,0,0,1,1) = 6%, P(0,1,0,0,0) = 3.65%, P(0,0,0,0,1) = 3.05%, and P(1,1,0,0,0) = 2.65%. Except for the non-carrying category (0,0,0,0,0), the pROH values in the RA patient group are greater than those for the control group (i.e., risk category). The finding that RA patients carry different ROHs partially reflects the genetic heterogeneity of RA.

### Copy number determination

**Figure 4:**
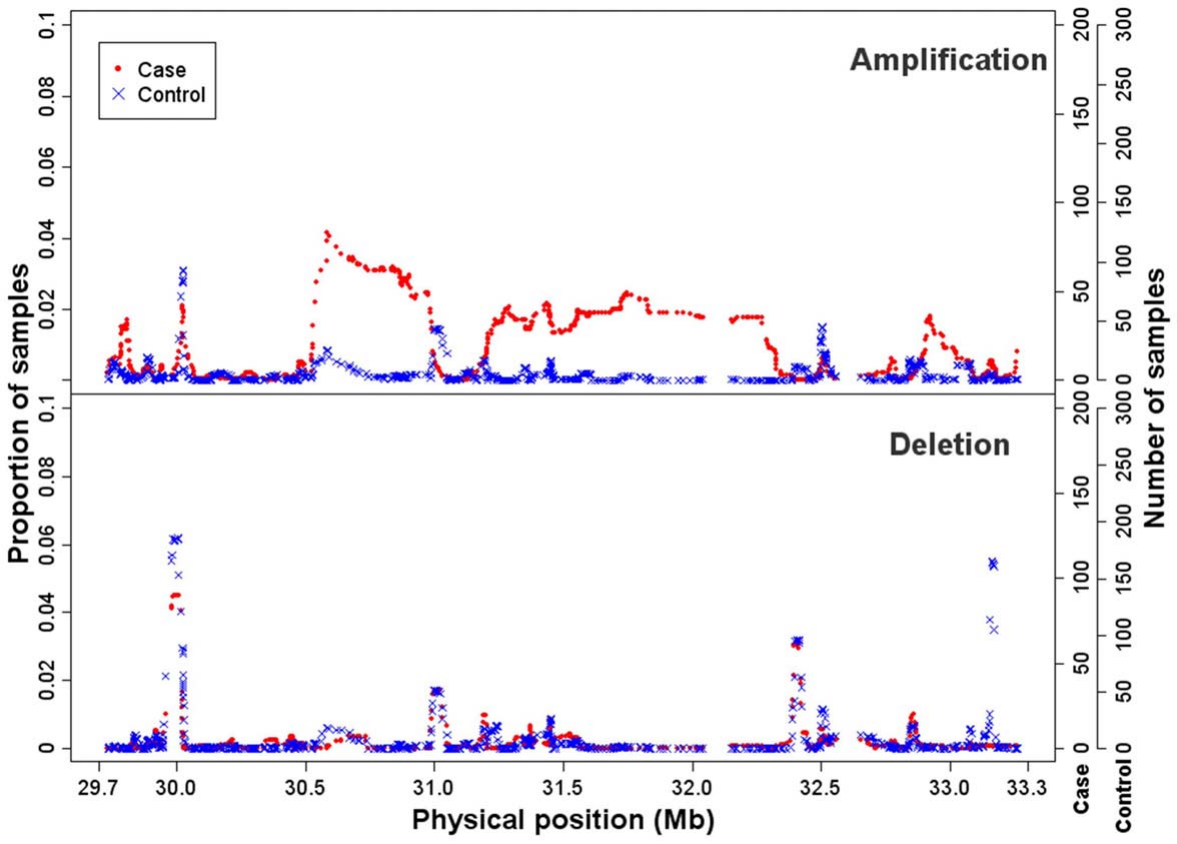
Gene amplifications and deletions in the MHC region. The fraction and numbers of RA patients and controls who carried amplifications and deletions in the MHC region are showed. The top and bottom panels show the results for the amplifications and deletions, respectively. In both panels, the results for the RA patients and controls are indicated as red dots and blue crosses, respectively. The fraction of RA patients and controls is shown on the left *y* axis, and the number of RA patients and controls is shown on the right *y* axis.

We detected genomic deletions (copy number <2) and amplifications (copy number >2) in the MHC regions of the 2,000 RA patients and 3,000 controls from the WTCCC study (Figure 4). Regarding the genomic deletions, no region in the RA patients was found to have a significantly greater proportion (a proportion difference >2%) of deletions than regions of the controls. Conversely, one region from the controls, rs1431403 (33,155,009 bp) to rs7764491 (33,168,818 bp), had a greater proportion of deletions than the regions from RA patients. The average proportion difference is 0.0509. Regarding genomic amplifications, three regions from the RA patients had a greater proportion of amplifications (a proportion difference >2%) than those of the controls. The three regions are rs2516670 (30,542,978 bp) to rs9295931 (30,977,693 bp), rs9295961 (31,275,477 bp) to rs9295967 (31,291,999 bp), and rs2736177 (31,694,073) to rs2299851 (31,826,581 bp), and the average proportion differences for the RA patient data minus the control data are 0.0282, 0.0201, and 0.0214, respectively.

## Discussion

Our study represents the first genome-wide homozygosity association scans for RA; we pinpointed important RA-associated ROHs in the MHC region and confirmed this region to be associated with RA [64], [65]. For the two ROHs, the window with the best prediction accuracy 62% is anchored by the SNP rs2027852. We validated the results derived from the WTCCC data by using the independently acquired NARAC data (Figure S2). Homozygosity disequilibrium was consistently found in the MHC region, for which the respective maximum values of −log_10_(p) for NARAC_100 (*W* = 100) and NARAC_200 (*W* = 200) are −log_10_(p) = 7.6973 and −log_10_(p) = 7.1334, respectively, which are highly significant values.

The SNP rs2027852 is flanked by *HLA-DRB6* and *HLA-DRB1*. The *HLA-DRB1*-shared epitope is an important determinant of RA susceptibility [10]. Associations between *HLA-DRB1* and RA susceptibility [10], [11], [12], [13], [66], [67] and between *HLA-DRB1* and the severity of RA [68], [69] have been made. In addition to *HLA-DRB1*, a second relevant ROH includes *HLA-DPA1* and *HLA-DPB1*. Previous studies produced inconclusive results concerning the relationship between RA and *HLA-DPA1* and *HLA-DPB1* [70], [71], [72]. Despite the evidence of statistical significance supported by this study, more functional studies are necessary to re-confirm the genetic associations with RA.

We found that the observed homozygosity disequilibrium in the MHC region is not explained by mechanisms associated with hemizygous deletion because our copy number analysis found only a very small proportion of the samples had acquired DNA deletions in the MHC region (Figure 4). The RA-related ROHs probably were not generated from copy-neutral chromosomal aberrations, e.g., uniparental disomy and loss of heterozygosity, because such chromosomal abnormalities often result in severe inherited disorders and cancers, which the patients of the study did not have. Inbreeding, as the cause of the homozygosity disequilibrium, also seems unlikely as the patients were not an inbred population(s).

Selective sweep, a type of natural selection, seems to be a plausible mechanism for the appearance of homozygosity disequilibrium in general population [40]. Homozygosity disequilibrium in the MHC region, which has been shown to contain the important functional genes related to RA and other autoimmune diseases [64], [65], [73],[74], results in a loss of genetic diversities and thereby influences quantitative and/or qualitative alternations of expression profiles. Some studies have found that autoimmunity susceptibility genes are positively selected in RA [75], [76], [77], [78]. Selected alleles accumulate in the gene pool over time and consequently increase the probability of generating an ROH. Genomic regions with a small recombination fraction and a large LD tend to contain even more ROHs than do regions with large recombination fractions or a small LD; for example, the time necessary for a region to be affected by selective pressure is so short that a limited number of recombinations prevents a rapid decay of LD and thereby promotes the occurrence of ROHs [39]. For type-1 diabetes, a relevant study has also pointed out significant SNP identity and conserved extended haplotypes in the MHC region [44]. That and our study reinforce the idea that natural selection may be critical to maintaining functionally important genes [79] and susceptibility to complex diseases [80].

Our study attempted to tackle several difficulties associated with homozygosity association mapping, which is defined as the identification of ROHs associated with a given disease. However, the observed, extended homozygosity may contain a run of homozygotes, hemizygotes, or a combination of both, and the different types of runs may reflect different genetic mechanisms associated with a disease. For genotype-based homozygosity association mapping, it is difficult to distinguish the differences between true homozygosity (a homozygous run) and spurious homozygosity (a hemizygous run) [81], [82]. Therefore, we employed genotype-based homozygosity association scans and intensity-based copy-number characterization to discriminate between copy-neutral homozygosity and deletion-induced hemizygosity for the RA-associated ROHs. Additionally, missing genotypes or heterozygous calls that arise from genotyping errors or recent mutations may interrupt a homozygous run (imperfect ROH). The genome-wide homozygosity association mapping used in this study overcame these obstacles by imputing missing genotypes and correcting for the modest heterozygous interference with the use of a local polynomial fit [53].

The required minimum power value and sample size for genome-wide homozygosity association mapping for complex diseases have not been explicitly determined in previous studies [81]. Our simulations provided an objective assessment of how the values for the power and the number of samples affect the results, and the results for the simulations suggest that we used sufficient sample numbers to attain reasonable statistical power to detect RA-associated ROHs in this study. In contrast to a single-SNP recessive model, the homozygosity association tests provided by LOHAS and ROH programs are multilocus analysis methods. The two multilocus methods make use of genetic information from extent of homozygosity, which is a function of LD, recombination fraction, and population history [40]. Recessive-acting disease alleles in an ROH predisposing to a disease are accumulated and made use to elevate the low power of a single-SNP analysis due to rare disease alleles at single SNPs.

Population substructure/admixture is an important confounding factor in genome-wide case-control association studies. Ignoring the difference of genetic substructure/admixture in case and control groups may lead to false-positive findings. We thus also performed genome-wide homozygosity association test with an adjustment for population substructure/admixture using principal components. We regressed the homozygosity intensity estimates from LOHAS software [53] on case/control disease status and the first 10 principal components from EIGENSTRAT software [83] to validate genetic association we identified in the MHC region. We found that genetic association between the identified ROHs in the MHC region and RA disease status remained very significant after taking population substructure/admixture into account (Figure S3). The maximum −log_10_(p) values for the scans were 28.4155 for WTCCC_100, 23.1904 for WTCCC_150, and 14.6061 for WTCCC_200 in the first peak region and 8.6160 for WTCCC_100, 7.5250 for WTCCC_150, and 7.4240 for WTCCC_200 in the second peak region. The results explain that our findings in the MHC region are valid and robust to population substructure/admixture.

RA-associated ROHs identified by LOHAS software was also evaluated by a second homozygosity association method. ROH program [40], which has been integrated into HelixTree software (HelixTree, Inc.), was run to examine homozygosity association in the MHC region. Several parameter combinations for defining an ROH were considered in the analysis using ROH program. At the Bonferroni significance level, two significant RA-associated ROHs identified by LOHAS software were validated by ROH program (Figure S4).

In conclusion, our genome-wide homozygosity association study used high-density SNP array data to provide an alternative method to an allelic association study for mapping RA-susceptibility genes. Excess ROHs were found in the MHC regions of RA patients compared with those of controls, which uncovered a recessive component and missing heritability for RA and possibly other autoimmune diseases.

## Materials and Methods

### Study materials

We used SNP data from the WTCCC [18] that was obtained from 1,999 RA patients and 3,002 controls. Of the control samples, 1,502 were from the 1958 British Birth Cohort study and 1,500 were from the UK Blood Service. All samples were genotyped using the Affymetrix 500K SNP GeneChip system (Affymetrix Inc., Santa Clara, CA, USA). Genotypes were called using the genotype-calling algorithm, CHIAMO [18]. Samples from 868 RA patients and 1,194 normal controls participating in the NARAC [17] were used to independently validate the results of the WTCCC data. All samples were genotyped using the Infinium HumanHap550 SNP BeadChip system (Illumina Inc., San Diego, CA, USA). Genotypes were called with the genotyping module of BeadStudio. All samples passed a quality control examination. The SNP and gene annotation information including the physical positions and the associated genes were taken from the NCBI dbSNP Build 123.

### Statistical methods

A genome-wide non-parametric association test was applied to map regions of homozygosity disequilibrium in the genomes of the RA patients. Given a target SNP (anchor) on a chromosome, a window containing the target SNP and *W*-1 nearest neighbor SNPs was constructed. Windows were slid along the chromosomes. For the genomes of each individual studied and for each window, a homozygote intensity (fraction) of SNPs was estimated by non-parametric local polynomial fitting [84] with a tricubic weight function. Dependent variable in the local regression is the homozygous/heterozygous states of SNPs and independent variable is physical position of the SNPs [53]. Then, in each window, the estimated homozygote intensities for each individual were compared with the median homozygote intensities for all patient and control samples to calculate the Kullback-Leibler distance [85]. The larger the distance was, the greater the fraction of homozygous SNPs. A Wilcoxon rank sum test [86] was applied to compare the Kullback-Leibler distances for the patient and control groups, and then to identify windows/regions of greater median homozygote intensity for the patient genomes. The aforementioned procedures were executed by using LOHAS software (http://www.stat.sinica.edu.tw/hsinchou/genetics/loh/LOHAS.htm) [53]. Homozygote intensities in the regions of ROHs are used to predict RA status using statistical discriminant analysis [87] and a 10-fold cross-validation procedure. The average prediction accuracy of the fitted classifiers for the RA patients and the controls was calculated using the R package. Copy number analysis was performed using the Partek Genomics Suite (Partek, Inc.). Copy numbers were determined from the allele intensities with an adjustment for local GC content. Copy number alternations, including gene amplifications and deletions, were inferred by genomic segmentation for which the default parameters recommended by Partek were used.

## Supporting Information

Figure S1 **Distribution of the fraction of RA patients carrying ROHs in the two regions of homozygosity disequilibrium.** There are 60 anchor SNPs in the two regions that satisfy −log_10_(p)>8. The first region (ROH1) contains 26 anchor SNPs and 5 genes, and the second region (ROH2) contains 34 anchor SNPs and 4 genes. A red point is plotted if a patient carried an ROH at an anchor SNP; otherwise the space is blank. The relative positions of 9 genes in these 2 regions are shown, and the 5 anchor SNPs used to tag rs9268831, rs2027852, rs9272723, rs3077, and rs9277542 are also marked.
(PPT)

Figure S2 **Genome-wide homozygosity association scans for the NARAC and WTCCC data.** The values of −log_10_(p) at anchor SNPs for the two genome-wide homozygosity association scans, NARAC_100 (*W* = 100) and NARAC_200 (*W* = 200), are displayed. A genome-wide significance level of −log_10_(p) = 8 is marked by the purple, horizontal line. The results for the WTCCC_100 and WTCCC_200 scans are provided for comparison. Peaks with −log_10_(p) values above the significance line and signals that were consistently identified by the four scans were found in the MHC region on chromosome 6p21.3.
(TIF)

Figure S3 **Homozygosity association scans with an adjustment for population substructure/admixture in the MHC region for the WTCCC data using principal components.** The values of −log_10_(p) at the anchor SNPs for the three homozygosity association scans, WTCCC_100, WTCCC_150, and WTCCC_200, are displayed. WTCCC_100, blue line, circles; WTCCC_150, green line, crosses; WTCCC_200, orange line, triangles.
(TIFF)

Figure S4 **Homozygosity association scans in the MHC region for the WTCCC data using ROH program.** Two parameters for defining an ROH are required in ROH program: the minimum run length (Rmin) and the minimum number of samples (Smin). ROHs are disregarded if the number of homozygous SNPs is less than Rmin. SNPs are removed if the number of samples for which that SNP is a member of an ROH is less than Smin (the details can refer to the user guide of ROH program in HelixTree software). This analysis considered Rmin = {50, 100, 150, 200} and Smin = {100, 150, 200, 250, 300}. Moreover, 10,000 permutations were performed to evaluate genetic association between affection status of RA and ROHs in the MHC region. In each subfigure, the horizontal axis denotes physical position (unit: Mb) on chromosome 6 and the vertical axis denotes p-value (−log_10_ scale) from the homozygosity association test used in ROH program. A green solid line indicates a raw empirical p-value of homozygosity association tests from 10,000 permutations. Value of the raw empirical p-value is shown above the green line. Physical positions of starting and ending SNPs of an ROH are listed below the green line. A red dashed line indicates the Bonferroni significance level, i.e., 0.05/30 in this analysis. If no ROH was found under a certain parameter combination of Rmin and Smin, an empty subfigure is shown.
(TIFF)

Appendix S1 **Simulation studies for evaluating power of the homozygosity association test used in this paper.**
(DOC)

## References

[1] Lynn AH, Kwoh CK, Venglish CM, Aston CE, Chakravarti A (1995). Genetic Epidemiology of Rheumatoid Arthritis.. American Journal of Human Genetics.

[2] Firestein GS (2003). Evolving concepts of rheumatoid arthritis.. Nature.

[3] Klareskog L, Stolt P, Lundberg K, Kallberg H, Bengtsson C (2006). A new model for an etiology of rheumatoid arthritis.. Arthritis and Rheumatism.

[4] Mahdi H, Fisher BA, Kallberg H, Plant D, Malmstrom V (2009). Specific interaction between genotype, smoking and autoimmunity to citrullinated alpha-enolase in the etiology of rheumatoid arthritis.. Nature Genetics.

[5] Seldin MF, Amos CI, Ward R, Gregersen PK (1999). The genetics revolution and the assault on rheumatoid arthritis.. Arthritis and Rheumatism.

[6] Alamanos Y, Drosos AA (2005). Epidemiology of adult rheumatoid arthritis.. Autoimmunity Reviews.

[7] Sokka T, Abelson B, Pincus T (2008). Mortality in rheumatoid arthritis: 2008 update.. Clinical and Experimental Rheumatology.

[8] Jawaheer D, Seldin MF, Amos CI, Chen WV, Shigeta R (2001). A genomewide screen in multiplex rheumatoid arthritis families suggests genetic overlap with other autoimmune diseases.. American Journal of Human Genetics.

[9] Wordsworth P, Bell J (1991). Polygenic susceptibility in rheumatoid arthritis.. Annals of the Rheumatic Diseases.

[10] Gregersen PK, Silver J, Winchester RJ (1987). The shared epitope hypothesis. An approach to understanding the molecular genetics of susceptibility to rheumatoid arthritis.. Arthritis and Rheumatism.

[11] Ollier W, Thomson W (1992). Population genetics of rheumatoid arthritis.. Rheumatic Disease Clinics of North America.

[12] Jawaheer D, Li WT, Graham RR, Chen W, Damle A (2002). Dissecting the genetic complexity of the association between human leukocyte antigens and rheumatoid arthritis.. American Journal of Human Genetics.

[13] John S, Shephard N, Liu GY, Zeggini E, Cao MQ (2004). Whole-genome scan, in a complex disease, using 11,245 single-nucleotide polymorphisms: Comparison with microsatellites.. American Journal of Human Genetics.

[14] Begovich AB, Carlton VEH, Honigberg LA, Schrodi SJ, Chokkalingam AP (2004). A missense single-nucleotide polymorphism in a gene encoding a protein tyrosine phosphatase (PTPN22) is associated with rheumatoid arthritis.. American Journal of Human Genetics.

[15] Carlton VEH, Hu XL, Chokkalingam AP, Schrodi SJ, Brandon R (2005). PTPN22 genetic variation: Evidence for multiple variants associated with rheumatoid arthritis.. American Journal of Human Genetics.

[16] Plenge RM, Padyukov L, Remmers EF, Purcell S, Lee AT (2005). Replication of putative candidate-gene associations with rheumatoid arthritis in >4,000 samples from North America and Sweden: Association of susceptibility with PTPN22, CTLA4, and PADI4.. American Journal of Human Genetics.

[17] Plenge RM, Seielstad M, Padyukov L, Lee AT, Remmers EF (2007). TRAF1-C5 as a risk locus for rheumatoid arthritis - A genomewide study.. New England Journal of Medicine.

[18] The Wellcome Trust Case Control Consortium (2007). Genome-wide association study of 14,000 cases of seven common diseases and 3,000 shared controls.. Nature.

[19] Raychaudhuri S, Remmers EF, Lee AT, Hackett R, Guiducci C (2008). Common variants at CD40 and other loci confer risk of rheumatoid arthritis.. Nature Genetics.

[20] Stahl EA, Raychaudhuri S, Remmers EF, Xie G, Eyre S (2010). Genome-wide association study meta-analysis identifies seven new rheumatoid arthritis risk loci.. Nature Genetics.

[21] Hirschhorn JN, Daly MJ (2005). Genome-wide association studies for common diseases and complex traits.. Nature Reviews Genetics.

[22] Kurreeman FAS, Padyukov L, Marques RB, Schrodi SJ, Seddighzadeh M (2007). A candidate gene approach identifies the TRAF1/C5 region as a risk factor for rheumatoid arthritis.. PLoS Medicine.

[23] Thomson W, Barton A, Ke X, Eyre S, Hinks A (2007). Rheumatoid arthritis association at 6q23.. Nature Genetics.

[24] Remmers EF, Plenge RM, Lee AT, Graham RR, Hom G (2007). STAT4 and the risk of rheumatoid arthritis and systemic lupus erythematosus.. New England Journal of Medicine.

[25] Gregersen PK, Amos CI, Lee AT, Lu Y, Remmers EF (2009). REL, encoding a member of the NF-kappa B family of transcription factors, is a newly defined risk locus for rheumatoid arthritis.. Nature Genetics.

[26] Barton A, Thomson W, Ke X, Eyre S, Hinks A (2008). Rheumatoid arthritis susceptibility loci at chromosomes 10p15, 12q13 and 22q13.. Nature Genetics.

[27] Gregersen PK (2010). Susceptibility genes for rheumatoid arthritis - a rapidly expanding harvest.. Bulletin of the NYU Hospital for Joint Diseases.

[28] Cavenee WK, Dryja TP, Phillips RA, Benedict WF, Godbout R (1983). Expression of recessive alleles by chromosomal mechanisms in retinoblastoma.. Nature.

[29] Koufos A, Hansen MF, Copeland NG, Jenkins NA, Lampkin BC (1985). Loss of heterozygosity in 3 embryonal tumors suggests a common pathogenetic mechanism.. Nature.

[30] Yokota J, Wada M, Shimosato Y, Terada M, Sugimura T (1987). Loss of heterozygosity on chromosomes 3, 13, and 17 in small-cell carcinoma and on chromosome 3 in adenocarcinoma of the lung.. Proceedings of the National Academy of Sciences of the United States of America.

[31] Yamamoto G, Nannya Y, Kato M, Sanada M, Levine RL (2007). Highly sensitive method for genomewide detection of allelic composition in nonpaired, primary tumor specimens by use of affymetrix single-nucleotide-polymorphism genotyping microarrays.. American Journal of Human Genetics.

[32] Huie ML, Anyane-Yeboa K, Guzman E, Hirschhorn R (2002). Homozygosity for multiple contiguous single-nucleotide polymorphisms as an indicator of large heterozygous deletions: identification of a novel heterozygous 8-kb intragenic deletion (IVS7–19 to IVS15–17) in a patient with glycogen storage disease type II.. American Journal of Human Genetics.

[33] Broman KW, Weber JL (1999). Long homozygous chromosomal segments in reference families from the centre d'Etude du polymorphisme humain.. American Journal of Human Genetics.

[34] Li LH, Ho SF, Chen CH, Wei CY, Wong WC (2006). Long contiguous stretches of homozygosity in the human genome.. Human Mutation.

[35] Wang S, Haynes C, Barany F, Ott J (2009). Genome-wide autozygosity mapping in human populations.. Genetic Epidemiology.

[36] Nalls MA, Simon-Sanchez J, Gibbs JR, Paisan-Ruiz C, Bras JT (2009). Measures of autozygosity in decline: globalization, urbanization, and its implications for medical genetics.. PLoS Genetics.

[37] McQuillan R, Leutenegger AL, Abdel-Rahman R, Franklin CS, Pericic M (2008). Runs of homozygosity in European populations.. American Journal of Human Genetics.

[38] Sabeti PC, Varilly P, Fry B, Lohmueller J, Hostetter E (2007). Genome-wide detection and characterization of positive selection in human populations.. Nature.

[39] Sabeti PC, Reich DE, Higgins JM, Levine HZ, Richter DJ (2002). Detecting recent positive selection in the human genome from haplotype structure.. Nature.

[40] Lencz T, Lambert C, DeRosse P, Burdick KE, Morgan TV (2007). Runs of homozygosity reveal highly penetrant recessive loci in schizophrenia.. Proceedings of the National Academy of Sciences of the United States of America.

[41] Gibson J, Morton NE, Collins A (2006). Extended tracts of homozygosity in outbred human populations.. Human Molecular Genetics.

[42] Nothnagel M, Lu TT, Kayser M, Krawczak M (2010). Genomic and geographic distribution of SNP-defined runs of homozygosity in Europeans.. Human Molecular Genetics.

[43] Nalls MA, Guerreiro RJ, Simon-Sanchez J, Bras JT, Traynor BJ (2009). Extended tracts of homozygosity identify novel candidate genes associated with late-onset Alzheimer's disease.. Neurogenetics.

[44] Baschal EE, Aly TA, Jasinski JM, Steck AK, Noble JA (2009). Defining multiple common “completely” conserved major histocompatibility complex SNP haplotypes.. Clinical Immunology.

[45] Lander ES, Botstein D (1987). Homozygosity mapping: a way to map human recessive traits with the DNA of inbred children.. Science.

[46] Sheffield VC, Carmi R, Kwitekblack A, Rokhlina T, Nishimura D (1994). Identification of a Bardet-Biedl syndrome locus on chromosome 3 and evaluation of an efficient approach to homozygosity mapping.. Human Molecular Genetics.

[47] Christodoulou K, Tsingis M, Deymeer F, Serdaroglu P, Ozdemir C (1997). Mapping of the familial infantile myasthenia (congenital myasthenic syndrome type Ia) gene to chromosome 17p with evidence of genetic homogeneity.. Human Molecular Genetics.

[48] Parvari R, Hershkovitz E, Kanis A, Gorodischer R, Shalitin S (1998). Homozygosity and linkage-disequilibrium mapping of the syndrome of congenital hypoparathyroidism, growth and mental retardation, and dysmorphism to a 1-cM interval on chromosome 1q42–43.. American Journal of Human Genetics.

[49] Winick JD, Blundell ML, Galke BL, Salam AA, Leal SM (1999). Homozygosity mapping of the achromatopsia locus in the pingelapese.. American Journal of Human Genetics.

[50] Goldberg EK, Glendening JM, Karanjawala Z, Sridhar A, Walker GJ (2000). Localization of multiple melanoma tumor-suppressor genes on chromosome 11 by use of homozygosity mapping-of-deletions analysis.. American Journal of Human Genetics.

[51] Huggins R, Li LH, Lin YC, Yu AL, Yang HC (2008). Nonparametric estimation of LOH using Affymetrix SNP genotyping arrays for unpaired samples.. Journal of Human Genetics.

[52] Gunduz E, Gunduz M, Ali MA, Beder L, Tamamura R (2009). Loss of heterozygosity at the 9p21–24 region and identification of BRM as a candidate tumor suppressor gene in head and neck squamous cell carcinoma.. Cancer Investigation.

[53] Yang HC, Chang LC, Huggins RM, Chen CH, Mullighan CG (2011). LOHAS: loss-of-heterozygosity analysis suite.. Genetic Epidemiology.

[54] Campbell H, Rudan I, Bittles AH, Wright AF (2009). Human population structure, genome autozygosity and human health.. Genome Medicine.

[55] Yang TL, Guo Y, Zhang LS, Tian Q, Yan H (2010). Runs of homozygosity identify a recessive locus 12q21.31 for human adult height.. The Journal of Clinical Endocrinology & Metabolism.

[56] Campbell H, Carothers AD, Rudan I, Hayward C, Biloglav Z (2007). Effects of genome-wide heterozygosity on a range of biomedically relevant human quantitative traits.. Human Molecular Genetics.

[57] Purcell S, Neale B, Todd-Brown K, Thomas L, Ferreira MAR (2007). PLINK: A tool set for whole-genome association and population-based linkage analyses.. American Journal of Human Genetics.

[58] Curtis D, Vine AE, Knight J (2008). Study of regions of extended homozygosity provides a powerful method to explore haplotype structure of human populations.. Annals of Human Genetics.

[59] Zhang L, Yang W, Ying D, Cherny SS, Hildebrandt F (2011). Homozygosity mapping on a single patient-identification of homozygous regions of recent common ancestry by using population data.. Human Mutation.

[60] Yang HC, Lin HC, Huang MC, Li LH, Pan WH (2010). A new analysis tool for individual-level allele frequency for genomic studies.. BMC Genomics.

[61] Pfeifer D, Pantic M, Skatulla I, Rawluk J, Kreutz C (2007). Genome-wide analysis of DNA copy number changes and LOH in CLL using high-density SNP arrays.. Blood.

[62] Yang HC, Huang MC, Li LH, Lin CH, Yu ALT (2008). MPDA: Microarray pooled DNA analyzer.. BMC Bioinformatics.

[63] Wang K, Li M, Hadley D, Liu R, Glessner J (2007). PennCNV: an integrated hidden Markov model designed for high-resolution copy number variation detection in whole-genome SNP genotyping data.. Genome Research.

[64] Newton JL, Harney SMJ, Wordsworth BP, Brown MA (2004). A review of the MHC genetics of rheumatoid arthritis.. Genes and Immunity.

[65] Weyand CM, Goronzy JJ (2000). Association of MHC and rheumatoid arthritis. HLA polymorphisms in phenotypic variants of rheumatoid arthritis.. Arthritis Research.

[66] Wu CC, Shete S, Chen WV, Peng B, Lee AT (2009). Detection of disease-associated deletions in case-control studies using SNP genotypes with application to rheumatoid arthritis.. Human Genetics.

[67] Wordsworth BP, Lanchbury JSS, Sakkas LI, Welsh KI, Panayi GS (1989). HLA-DR4 subtype frequencies in rheumatoid arthritis indicate that DRB1 is the major susceptibility locus within the HLA class II region.. Proceedings of the National Academy of Sciences of the United States of America.

[68] Constantin A, Lauwers-Cances V, Navaux F, Abbal M, van Meerwijk J (2002). Collagenase-1 (MMP-1) and HLA-DRB1 gene polymorphisms in rheumatoid arthritis: a prospective longitudinal study.. The Journal of Rheumatology.

[69] Weyand CM, Hicok KC, Conn DL, Goronzy JJ (1992). The influence of HLA-DRB1 genes on disease severity in rheumatoid arthritis.. Annals of Internal Medicine.

[70] Begovich AB, Bugawan TL, Nepom BS, Klitz W, Nepom GT (1989). A specific HLA-DP beta allele is associated with pauciarticular juvenile rheumatoid arthritis but not adult rheumatoid arthritis.. Proceedings of the National Academy of Sciences of the United States of America.

[71] Carthy D, MacGregor A, Awomoi A, Rigby AS, Thomson W (1995). HLA-DPB1*0201 is associated with particular clinical features of rheumatoid arthritis.. Revue du rhumatisme (English ed).

[72] Yen JH, Chen JR, Tsai WJ, Tsai JJ, Liu HW (1995). HLA-DPB1 polymorphism in patients with rheumatoid arthritis in Taiwan.. The Journal of Rheumatology.

[73] Fernando MM, Stevens CR, Walsh EC, De Jager PL, Goyette P (2008). Defining the role of the MHC in autoimmunity: a review and pooled analysis.. PLoS Genetics.

[74] Ridgway WM, Fasso M, Fathman CG (1999). A new look at MHC and autoimmune disease.. Science.

[75] Datta SK (2000). Positive selection for autoimmunity.. Nature Medicine.

[76] Albani S, Keystone EC, Nelson JL, Ollier WE, La Cava A (1995). Positive selection in autoimmunity: abnormal immune responses to a bacterial dnaJ antigenic determinant in patients with early rheumatoid arthritis.. Nature Medicine.

[77] Kretz-Rommel A, Rubin RL (2000). Disruption of positive selection of thymocytes causes autoimmunity.. Nature Medicine.

[78] Limaye N, Belobrajdic KA, Wandstrat AE, Bonhomme F, Edwards SV (2008). Prevalence and evolutionary origins of autoimmune susceptibility alleles in natural mouse populations.. Genes and Immunity.

[79] Bersaglieri T, Sabeti PC, Patterson N, Vanderploeg T, Schaffner SF (2004). Genetic signatures of strong recent positive selection at the lactase gene.. American Journal of Human Genetics.

[80] Marigorta UM, Lao O, Casals F, Calafell F, Morcillo-Suarez C (2011). Recent human evolution has shaped geographical differences in susceptibility to disease.. BMC Genomics.

[81] Ku CS, Naidoo N, Teo SM, Pawitan Y (2011). Regions of homozygosity and their impact on complex diseases and traits.. Human Genetics.

[82] Peiffer DA, Le JM, Steemers FJ, Chang WH, Jenniges T (2006). High-resolution genomic profiling of chromosomal aberrations using Infinium whole-genome genotyping.. Genome Research.

[83] Price AL, Patterson NJ, Plenge RM, Weinblatt ME, Shadick NA (2006). Principal components analysis corrects for stratification in genome-wide association studies.. Nature Genetics.

[84] Loader C (1999). Local regression and likelihood.

[85] Kullback S, Leibler RA (1951). On information and sufficiency.. Annals of Mathematical Statistics.

[86] Wilcoxon F (1945). Individual comparisons by ranking methods.. Biometrics Bulletin.

[87] Hastie T, Tibshirani R, Buja A (1994). Flexible discriminant analysis by optimal scoring.. Journal of the American Statistical Association.

