# Supplementary figures and images for: A Genome-Wide Homozygosity Association Study Identifies Runs of Homozygosity Associated with Rheumatoid Arthritis in the Human Major Histocompatibility Complex
Source: PLoS One. 2012 Apr 20;7(4):e34840. doi: 10.1371/journal.pone.0034840 (PMC3335047; doi:10.1371/journal.pone.0034840)

## Slide 1
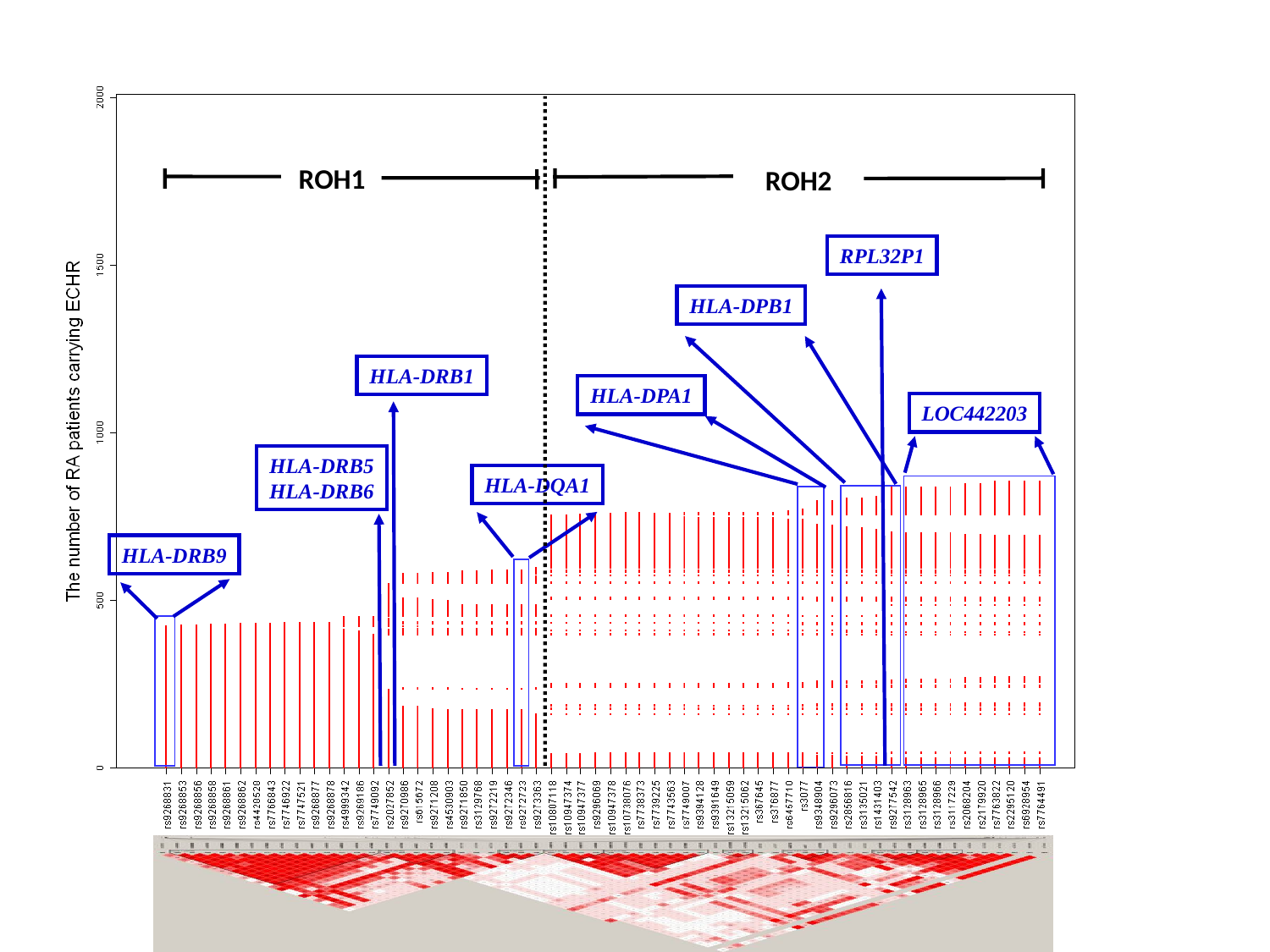

ROH1
ROH2
RPL32P1
HLA-DPB1
HLA-DRB1
HLA-DPA1
LOC442203
HLA-DRB5
HLA-DRB6
HLA-DQA1
HLA-DRB9
1
08/29/26

Supplement: Figure S1 — Distribution of the fraction of RA patients carrying ROHs in the two regions of homozygosity disequilibrium. There are 60 anchor SNPs in the two regions that satisfy −log10(p)>8. The first region (ROH1) contains 26 anchor SNPs and 5 genes, and the second region (ROH2) contains 34 anchor SNPs and 4 genes. A red point is plotted if a patient carried an ROH at an anchor SNP; otherwise the space is blank. The relative positions of 9 genes in these 2 regions are shown, and the 5 anchor SNPs used to tag rs9268831, rs2027852, rs9272723, rs3077, and rs9277542 are also marked. (PPT) [file pone.0034840.s001.ppt]

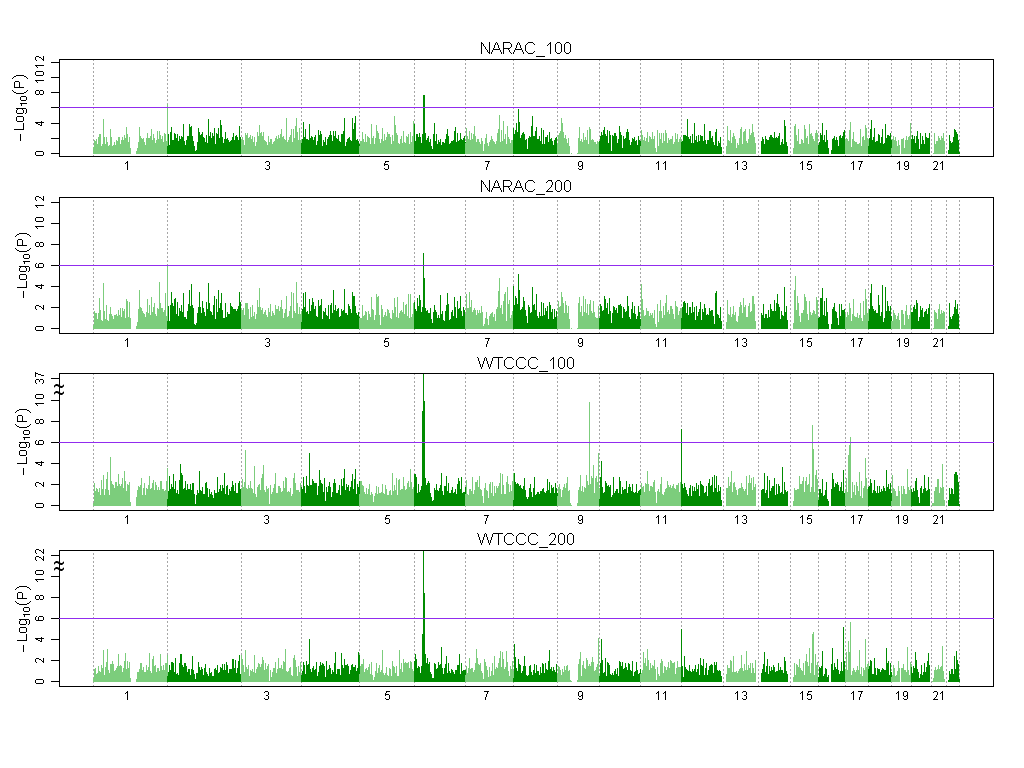

Supplement: Figure S2 — Genome-wide homozygosity association scans for the NARAC and WTCCC data. The values of −log10(p) at anchor SNPs for the two genome-wide homozygosity association scans, NARAC_100 (W = 100) and NARAC_200 (W = 200), are displayed. A genome-wide significance level of −log10(p) = 8 is marked by the purple, horizontal line. The results for the WTCCC_100 and WTCCC_200 scans are provided for comparison. Peaks with −log10(p) values above the significance line and signals that were consistently identified by the four scans were found in the MHC region on chromosome 6p21.3. (TIF) [file pone.0034840.s002.tif]

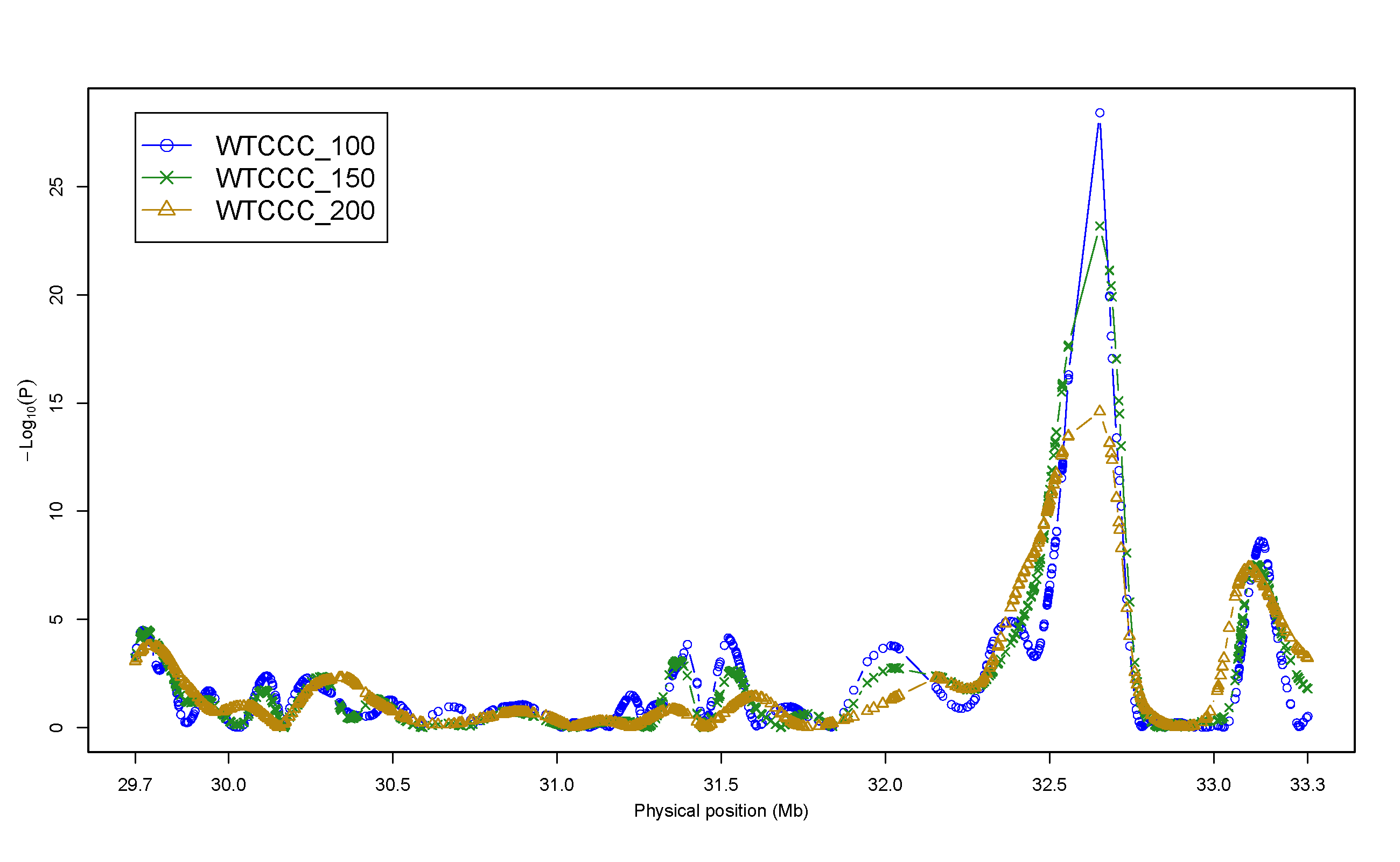

Supplement: Figure S3 — Homozygosity association scans with an adjustment for population substructure/admixture in the MHC region for the WTCCC data using principal components. The values of −log10(p) at the anchor SNPs for the three homozygosity association scans, WTCCC_100, WTCCC_150, and WTCCC_200, are displayed. WTCCC_100, blue line, circles; WTCCC_150, green line, crosses; WTCCC_200, orange line, triangles. (TIFF) [file pone.0034840.s003.tif]

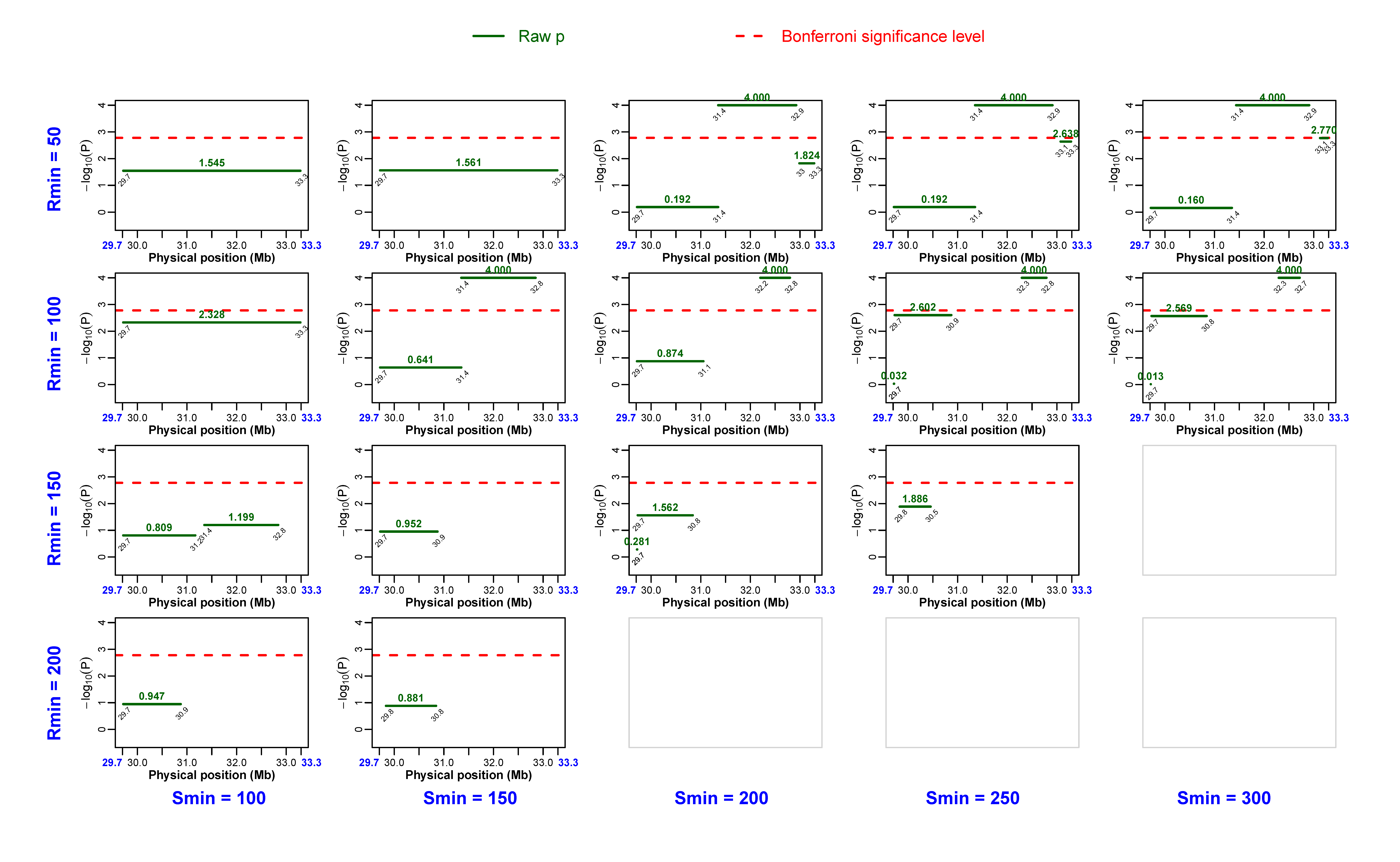

Supplement: Figure S4 — Homozygosity association scans in the MHC region for the WTCCC data using ROH program. Two parameters for defining an ROH are required in ROH program: the minimum run length (Rmin) and the minimum number of samples (Smin). ROHs are disregarded if the number of homozygous SNPs is less than Rmin. SNPs are removed if the number of samples for which that SNP is a member of an ROH is less than Smin (the details can refer to the user guide of ROH program in HelixTree software). This analysis considered Rmin = {50, 100, 150, 200} and Smin = {100, 150, 200, 250, 300}. Moreover, 10,000 permutations were performed to evaluate genetic association between affection status of RA and ROHs in the MHC region. In each subfigure, the horizontal axis denotes physical position (unit: Mb) on chromosome 6 and the vertical axis denotes p-value (−log10 scale) from the homozygosity association test used in ROH program. A green solid line indicates a raw empirical p-value of homozygosity association tests from 10,000 permutations. Value of the raw empirical p-value is shown above the green line. Physical positions of starting and ending SNPs of an ROH are listed below the green line. A red dashed line indicates the Bonferroni significance level, i.e., 0.05/30 in this analysis. If no ROH was found under a certain parameter combination of Rmin and Smin, an empty subfigure is shown. (TIFF) [file pone.0034840.s004.tif]
