# Supplementary material for: A Genome-Wide Homozygosity Association Study Identifies Runs of Homozygosity Associated with Rheumatoid Arthritis in the Human Major Histocompatibility Complex
Source: PLoS One. 2012 Apr 20;7(4):e34840. doi: 10.1371/journal.pone.0034840 (PMC3335047; doi:10.1371/journal.pone.0034840)
Supplement: Appendix S1 — Simulation studies for evaluating power of the homozygosity association test used in this paper. (DOC) [file pone.0034840.s005.doc]

**Appendix S1 –** Simulation studies for evaluating power of the homozygosity association test used in this paper.

We conducted simulation studies to evaluate power of the homozygosity association test used in this paper. Data were generated to mimic the practical scenarios in our WTCCC data analysis with 2,000 RA patients and 3,000 normal controls. For normal controls, homozygosity intensities were generated following a uniform distribution of 0.7$\pm$0.02, similar to the empirical distribution of normal controls in the WTCCC data. For RA patients without carrying an RA-associated ROH, the genomic pattern of homozygous intensities of these patients is similar to normal controls. For RA patients who carried an RA-associated ROH, homozygosity intensity data were generated by considering the following three parameters: (1) the number of SNPs in the RA-associated ROH (*L*), *L* = 100, 150 and 200, (2) the proportion of RA patients who carried the RA-associated ROH (*δ*), i.e., the effect size in our simulation study, *δ* = 0.1, 0.2 and 0.3, and (3) the fraction of non-homozygous SNPs in the RA-associated ROH of RA patients who carried the RA-associated ROH (*ε*), *ε* = 0, 0.1 and 0.2. For each simulation condition, 1,000 simulation replications were performed. Three window sizes (*W*) were considered when we estimated homozygosity intensities for every individual by using LOHAS software: *W* = 100, 150 and 200. The homozygosity association test used in this paper was performed by using LOHAS software. We calculated the proportion of rejecting null hypothesis among 1,000 simulation replications to yield power.
